# Longitudinal dynamics of plasma bile acids and their associations with physiological parameters and fecal microbiome during the transition period in dairy cows

**DOI:** 10.5713/ab.24.0628

**Published:** 2025-02-27

**Authors:** Feixiang Fan, Liang Chen, Huizeng Sun, Jianxin Liu, Kailun Yang, Fengfei Gu

**Affiliations:** 1 College of Animal Sciences, Xinjiang Key Laboratory of Herbivorous Nutrition for Meat & Milk, Xinjiang Agricultural University, Urumqi, China; 2 Institute of Dairy Science, College of Animal Sciences, Zhejiang University, Hangzhou, China

**Keywords:** Hindgut Microbiota, Perinatal Dairy Cow, Ursodeoxycholic Acid

## Abstract

**Objective:**

The aim of this study is to investigate the dynamic changes of plasma bile acids (BA) and their correlations with physiological parameters and fecal microbiome in transitional dairy cows.

**Methods:**

Twenty multiparous dairy cows were selected, the blood and fecal samples were collected on d −21, −7, +7, and +21 of calving. The targeted metabolome and 16s rDNA ampicon sequencing were utilized to identify BA profiles and fecal microbial composition, respectively.

**Results:**

A total of 32 BAs were found, comprising 9 primary BAs (PBA) and 23 secondary BAs (SBA). Majority of the PBAs (7 out to 9) and SBAs (15 out to 23) exhibited significant increases postpartum compared to prepartum levels. Notably, ursodeoxycholic acid, taurocholic acid and 7-ketodeoxycholic acid showed higher importance. Correlation analysis showed the BAs concentrations positively correlated with the concentrations of aspartate aminotransferase, total antioxidant capacity, and glutathione peroxidase, while exhibiting substantial negative correlation with triglyceride concentrations. A decline in bacterial alpha diversity in postpartum and significantly different β-diversity were observed. Furthermore, 30 distingtive genera were identified over the transition period. Among these, six and eleven biomarkers such as *Alistipes* and *Ruminococcaceae_UCG*_*014* were identified at +7 d and +21 d, respectively. Furthermore, the abundances of choloylglycine hydrolase and 7-alpha-hydroxysteroid dehydrogenase which are involved in SBA biosynthesis were significantly higher postpartum as determined by PICRUSt2 analysis over the transition period.

**Conclusion:**

The BA profile and concentrations underwent significant changes during the transition period in dairy cows and these changes are closely related to the periparturient health of the cows. Ursodeoxycholic acid and *Alistipes* was identified as the pivotal BA and microbial genus. Our study elucidates these metabolic processes, providing useful insights into strategies for enhancing the nutrition and well-being of perinatal dairy cows.

## INTRODUCTION

The transition period is a critical stage for dairy cows, during which the risk of diseases increases [1]. It has been reported that 30% to 50% of dairy cows experience health disorders, and approximately 75% of infectious diseases and metabolic disorders in dairy cows occur during this stage [2,3]. This is partly attributed to the complex metabolic adaptations during the transition period, when the cows are challenged by nutrient supply to meet the demands of calving and the onset of milk production [4]. Therefore, the metabolic changes during the transition period are as important and challenging as calving.

Bile acids (BAs), consisting of a polar carboxylate side chain and a nonpolar steroid carbon skeleton, are synthesized into primary BAs (PBAs) in the liver from cholesterol [5]. Meanwhile, gut microbiota modify PBAs to produce secondary BAs (SBAs) through dehydroxylation, deconjugation, or both [6]. Traditionally, BAs have been recognized for their roles in facilitating lipid and fat-soluble vitamin digestion and absorption [7,8]. Recently, numerous studies have unveiled their multifaceted functions in maintaining host metabolism [9], immune functions [10], intestinal health, and gut microbial homeostasis [11]. In terms of animal industry, BAs and related additives have been extensively employed in monogastric animals to improve growth, development, and health status [12–14]. However, the requirement and effects of BAs in dairy cows are not well understood.

For transitional dairy cows, the metabolic changes of BAs and their subsequent effects warrant attention. It is widely recognized that the profile and concentration of BAs are intricately associated with hepatic and intestinal microbiota [15]. However, the liver of postpartum dairy cows often suffers from metabolic stress and may develop varying degrees of hepatic steatosis, attributed to negative energy balance (NEB) and subsequent adaptive responses [16]. Our previous research revealed substantial alterations in the relative abundance, microbial composition, and functions of fecal microbiota over the transition period in dairy cows [17]. Furthermore, postpartum dairy cows experience adipose mobilization due to NEB [18]. Overall, it can be reasonably assumed that the profiles and contents of BAs change significantly due to variations in hepatic, gut microbiota, and lipid metabolism in transitional dairy cows. However, there is currently a dearth of systematic research on alterations in BAs during the transition period in dairy cows, with only sporadic discussions available [19]. On the other hand, a subsequent inquiry is the potential additional ramifications of the adapting alterations in BA on transitional dairy cows. Many studies have demonstrated the pivotal role of specific BAs such as ursodeoxycholic acid (UDCA) or bile salts in regulating inflammation [20] and oxidative stress [21]. Our previous study has also established a link between BAs and immunosuppression in dairy cows [22]. Hence, the relationships between dynamic changes in BAs and health status, such as inflammation and oxidative stress, are areas of significant interest in dairy cows during the perinatal period, yet remain incompletely understood.

Thus, the objectives of this study are to unravel dynamic alterations in peripheral BA profiles and concentrations in dairy cows over the transition period and to decipher the intricate associations between changes in BA and physiological metabolism and gut microbiome. Capturing the dynamic shifts in BA metabolism during this exceptional transition window and delineating its connections with health outcomes and gut microbiota hold great potential for improving transitional care in dairy cows.

## MATERIALS AND METHODS

### Animals and experimental design

The animal experimental design was described in our companion study [17] with minor modification. In brief, twenty multiparous healthy Chinese Holstein dairy cows at d 28 before parturition (body weight: 690 kg, standard deviation [SD] = 50; Parity: 1.93, SD = 1; BCS: 3.21, SD = 0.41) with similar body weight were selected. The definition of a healthy dairy cow includes two aspects: first, the cow was not experienced any clinical diseases within one month before the dry period according to veterinary records; second, during the trial, the cow is free from any clinical disease as confirmed by veterinary. The first 7 d (wk -4 relative to parturition) was the adaptation period. Ingredients of the basal diet before calving and after calving have been reported by Zhu et al [17] (Supplement 1). The experiment ended at d 21 after calving. Throughout the whole trial period, cows were housed in a barn with individual tie stalls and had free access to water. Prolonged labor lasting over 4 hours without urination and with the amniotic sac ruptured is considered dystocia, and assistance from manual delivery is required. High-risk cattle including those with retained placenta, twin calves, stillbirth, and birth canal tears, are closely monitored, and the characteristics of uterine discharge and the health status of the cattle are carefully recorded. If watery foul-smelling or purulent foul-smelling uterine discharge is present, uterine inflammation is diagnosed.

### Blood and fecal sampling

Blood samples were collected into tubes containing an anticoagulant (heparin lithium) from the coccygeal vein of cows at 3 hours after the morning feeding at d −21, −7, 7, and 21 relative to the calving date. The samples were centrifuged at 3,000×g for 15 min at 4°C to collect plasma and then frozen at −20°C until subsequent analysis. An autoanalyzer 7020 instrument (Hitachi High-Technologies Corporation, Tokyo, Japan) was used to quantify concentrations of glucose (GLU; #ZH2079T), non-esterified fatty acids (NEFA; #ZH2045Z), β-hydroxybutyrate (BHBA; #ZH2029T), blood urea nitrogen (#ZH2017S), cholesterol (#ZH2040Z), triglycerides (TG; #ZH2039Z), albumin (#ZH2013G), alanine aminotransferase (ALT; #ZH2001G), aspartate aminotransferase (AST; #ZH2002G), total protein (TP; #ZH2012G), superoxide dismutase (SOD; #ZH2058F) and creatinine (#ZH2020S2) in plasma with commercial kits (Ningbo Medical System Biotechnology Co., Ltd., Ningbo, China). Likewise, commercial assay kits from Nanjing Jiancheng Bioengineering Institute (Nanjing, China) were used to quantify concentrations of plasma catalase (#A007-1-1), total antioxidant capacity (T-AOC; #A015-2-1), glutathione peroxidase (GSH-px; #A005-1-2), malondialdehyde (MDA; #A003-1-2), haptoglobin (HPT; #H136), amyloid (#H134) and ceruloplasmin (#A029-1-1) following the manufacturer’s instructions. Finally, plasma total oxidative status (TOS; #KC5100, Bensheim, Germany) was measured with commercial assay kits following manufacturer’s instructions [23]. The oxidative status indicator (OSI) was defined as the ratio of TOS to T-AOC [24,25]. Rectal fecal samples were collected before the morning feeding at 6:00 am using sterilized gloves and stored in 50 mL frozen storage tubes.

### Profiling of blood bile acids

Profiling of BA was conducted according to a method described by Hu et al [26]. Briefly, plasma (100 μL) was directly incubated with precooled methanol and internal standard. After centrifuging at 14,000×g for 15 min, the supernatants were collected for vacuum drying and then resuspended in 100 μL methanol:water (1:1, v/v). Analyses were performed using an UHPLC (Waters Ltd., Milford, MA, USA) coupled online to 5500 QTRAP Mass Spectrometry (AB SCIEX, Framingham, MA, USA). The peak area and retention time were generated by Multiquant software. The internal standards of BAs were used to correct the retention time and to identify metabolites.

### Microbial DNA extraction, sequencing, and pre-processing analysis

Microbial DNA from fecal samples was extracted using the E.Z.N.A. Stool DNA Kit (#D4015; Omega, Inc., Norcross, GA, USA). The universal 16S primers corresponding to the V3-V4 region (341F: 5’-CCTACGGGNGGCWGCAG-3’; 805R: 5’-GACTACHVGGGTATCTAATCC-3’) were used to amplify the bacterial 16S rRNA gene fragments. Purification and quality testing were performed using AMPure XT beads (Beckman Coulter Genomics, Danvers, MA, USA) and Qubit (Invitrogen, Waltham, MA, USA). Polymerase chain reaction (PCR) products were then pooled together and sequenced on an Illumina NovaSeq PE250, which was provided by LC-Bio Technology Co., Ltd, Hangzhou, China. The FLASH (V1.2.8) was applied to demultiplex the raw sequence data into paired-end fastq files. Alpha and beta diversities were calculated using QIIME2 [27]. The adonis test was used to determine significant differences caused by different categories of feed and host related metabolites. The significant differential microbiota was identified by LEfSe analysis. The functional components of the microbiome were predicted by PICRUSt2 program [28]. Among the twenty cows, eleven cows microbial data were shared with our companion study [17].

### Correlation analysis

The Spearman’s rank correlation analysis (R packages Hmisc v 4.6.0) was used to determine the associations among the BAs, microbiota and plasma parameters in current study. The P-values were generated using the t or F distributions, and p<0.05 was regarded as significantly correlation.

### Statistical analysis

The plasma BA and physiologic parameters data were analyzed using the PROC MIXED procedure with repeated measurement in SAS software (version 9.4), and covariance type AR (1) was selected. The model included the fixed effect of week and cow within the week as a random effect The experimental results were reported as least squares means, and the differences were separated using the PDIFF option in the LSMEANS treatment. Significance was declared at p≤0.05, and 0.05<p≤0.10 was considered a trend.

## RESULTS

### Dynamics of plasma bile acids concentration and relative proportion during transition period

In total, 32 BAs including 9 PBAs and 23 SBAs were identified from 80 plasma sample within 4 time points throughout the transition period, and the plasma concentration of BAs is presented in Supplement 2. Most of PBAs (7 out of 9) and SBAs (15 out of 23) showed significant differences (p<0.05) during the transition period, except for the taurochenodeoxycholic acid (TCDCA), tauroursodeoxycholic acid (TUDCA), β-muricholic acid (β-MCA),taurolithocholic acid (TLCA), taurohyodeoxycholic acid (THDCA), glycohyodeoxycholic acid (GHDCA), glycoursodeoxycholic acid (GUDCA) and ω-MCA.

**Figure 1:**
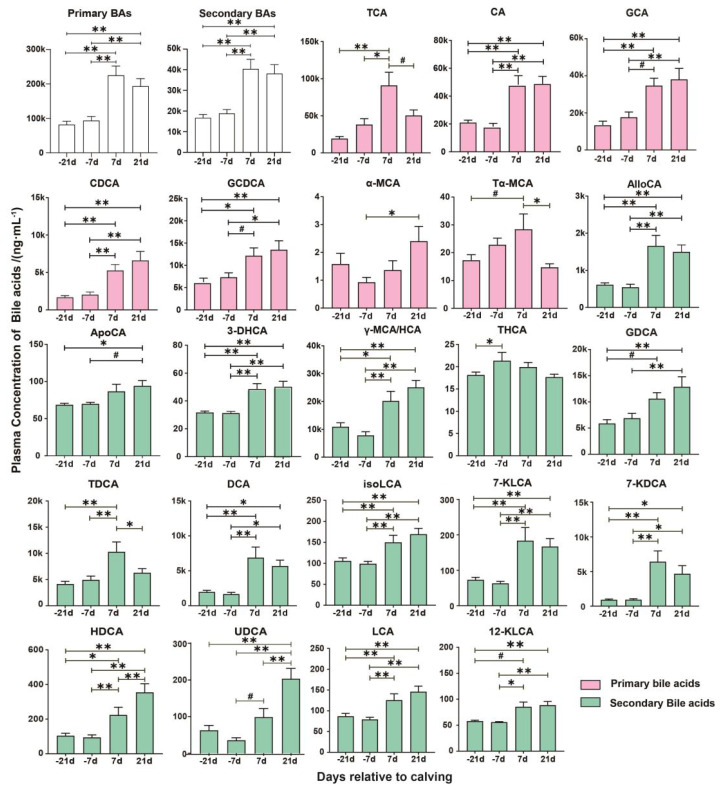
Concentrations of the plasma bile acids in dairy cows during the transition period. Only the bile acids with significant temporal differences are presented. * p<0.05, ** p<0.01, # 0.05<p<0.10. BA, bile acids; TCA, taurocholic acid; CA, cholic acid; GCA, glycocholic acid; CDCA, chenodeoxycholic acid; GCDCA, glycochenodeoxycholic acid; α-MCA, α-muricholic acid; Tα-MCA, tauro α-muricholic acid; AlloCA, allocholic acid; ApoCA, apocholic acid; 3-DHCA, 3-dehydrocholic acid; γ-MCA/HCA, γ-muricholic acid/hyocholic acid; THCA, taurohyocholic acid; GDCA, glycodeoxycholic acid; TDCA, taurodeoxycholic acid; DCA, deoxycholic acid; isoLCA, isolithocholic acid; 7-KLCA, 7-ketolithocholic acid; 7-KDCA, 7-ketodeoxycholic acid; HDCA, hyodeoxycholic acid; UDCA, ursodeoxycholic acid; LCA, lithocholic acid; 12-KLCA, 12-ketolithocholic acid.

Overall, total PBAs and SBAs significantly increased postpartum (p<0.01). Taurocholic acid (TCA) concentration peaked at d +7 compared to other time points (p<0.01), with no significant difference between d+21 and prepartum. At d +7, tauro α-MCA (Tα-MCA) was notably higher than at d +21 (p<0.01) and tended to be higher than at d −21 (p = 0.06). Conversely, α-MCA significantly increased at d +21 compared to −7 d (p<0.01), with no significant differences at other time points. Other PBAs, including cholic acid (CA), glycocholic acid (GCA), glycochenodeoxycholic acid (GCDCA), chenodeoxycholic acid (CDCA), all significantly increased postpartum. Regarding SBAs, apocholic acid (ApoCA) was significantly higher at d +21 than at d −21(p<0.01) and tended to be higher than at d −7 (p = 0.06). Taurohyocholic acid (THCA) notably increased at d −7 compared to d −21 (p<0.01), with no differences observed at other time points, glycodeoxycholic acid (GDCA) gradually increased, peaking at d +21 when significantly higher than at d −21 and d −7 (p<0.01). Taurodeoxycholic acid (TDCA) gradually increased, peaking at d +7, with significant differences compared to other time points. The UDCA concentration peaked at d +21, significantly higher than at d −21, −7, and +7 (p<0.01). Other SBAs content also significantly increased postpartum (Figure 1).

**Figure 2:**
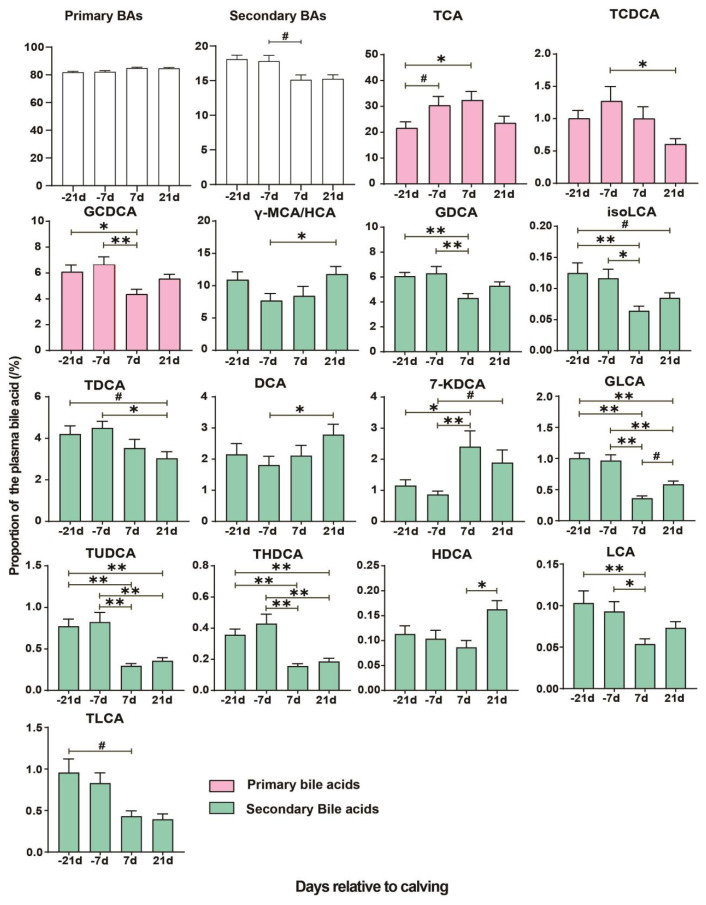
Proportions of the plasma bile acids in dairy cows during the transition period. Only the bile acids with significant temporal differences are presented. * p<0.05, ** p<0.01, # 0.05<p<0.10. BA, bile acids; TCA, urocholic acid; TCDCA, taurochenodeoxycholic acid; GCDCA, glycochenodeoxycholic acid; γ-MCA/HCA, γ-muricholic acid/hyocholic acid; GDCA, glycodeoxycholic acid; isoLCA, isolithocholic acid; TDCA, Taurodeoxycholic acid; DCA, deoxycholic acid; 7-KDCA, 7-ketodeoxycholic acid; GLCA, glycolithocholic acid; TUDCA, tauroursodeoxycholic acid; THDCA, Taurohyodeoxycholic acid; HDCA, Hyodeoxycholic acid; LCA, lithocholic acid.

The changes in the relative proportion of BAs are depicted in Supplement 3 and the Figure 2. While the proportion of total PBA remained stable during the perinatal period, the total SBA tended to be lower at d +7 compared to −7 d (p = 0.07). However, temporal differences in 3 PBAs (TCA, TCDCA, GCDCA) and 12 SBAs were observed. Specifically, the proportion of TCA followed its concentration changes, with significantly higher proportions at d −7 and +7 compared to −21 d (p<0.01). Significant differences between −7 d and +21 d were observed in the proportion changes of γ-MCA/HCA and TCDCA, but the trend was converse. For GCDCA, the proportion at d +7 was significantly lower than at d −21 and −7 (p<0.05). In terms of dynamic changes in SBA proportion, the proportion of 8 SBAs (GDCA, isoLCA, TDCA, GLCA, TUDCA, THDCA, LCA, TLCA) decreased significantly postpartum compared to prepartum. Conversely, the proportion of 7-ketodeoxycholic acid (7-KDCA) increased significantly postpartum (p<0.05). At d +21, the proportion of deoxycholic acid (DCA) and hyodeoxycholic acid (HDCA) was significantly higher than at d −7 (p<0.01) and +7 (p<0.01), respectively.

### Correlations of plasma bile acids with the blood parameters of the dairy cows during transition period

**Figure 3:**
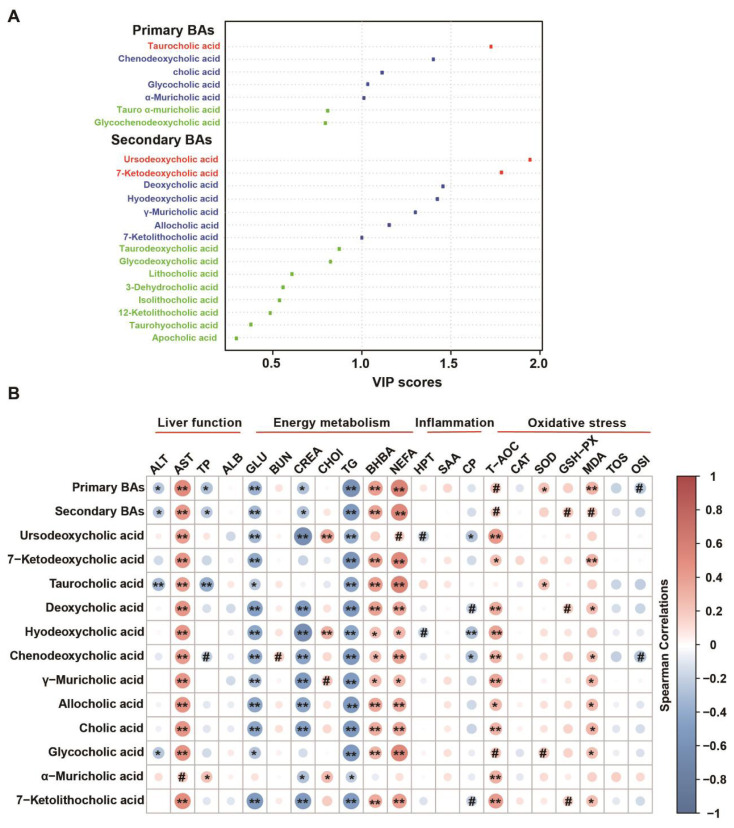
The important bile acid species and its associations with physiological metabolism in dairy cows during the transition period. (A) The variable importance in the projection analysis of the bile acids that with significant temporal differences. (B) The spearman correlations of the important bile acids with the plasma paremeters including liver functions, energy metabolism, inflammation and oxidative stress. * p<0.05, ** p<0.01, # 0.05<p<0.10. BA, bile acids; ALT, alanine aminotransferase; AST, aspartate aminotransferase; TP, total protein; ALB, albumin(A); GLU, glucose; BUN, blood urea nitrogen; CREA, creatinine; CHOI, cholesterol; TG, triglyceride; BHBA, β-hydroxybutyrate; NEFA, non-esterfied ftyacid; HPT, haptoglobin; SAA, serum amyloid A; CP, ceruloplasmin; T-AOC, total antioxidant capacity; CAT, catalase; SOD, superoxide dismutase; GPX-PX, glutathione peroxidase; MDA, malondialdehyde; TOS, total oxidant Status; OSI, TAS/TOS.

The variable importance in the projection (VIP) values were calculated to evaluate the importance of each BAs with time differences during the transition period, as shown in Figure 3A. Twelve BAs including 5 PBAs and 7 SBAs with VIP>1 and designated as key BAs. Among which, the TCA, UDCA, and 7-KDCA, exhibited higher VIP scores (VIP>1.5), with UDCA having the highest VIP score (VIP = 1.95).

The Spearman correlations between the total PBAs, SBAs and key BAs and plasma parameters refer to liver function, energy metabolism, inflammation and oxidative stress were presented in Figure 3B. The results showed that AST was positively correlated with total PBAs and SBAs, and most of the key BAs significantly (p<0.05) except for α-MCA. Conversely, ALT and TP showed negative correlations with total PBAs, SBAs, TCA significantly (p<0.05). The GCA is significantly negatively correlated with ALT (p = 0.03). Among the key BAs, the strongest correlations were observed with indicators of energy metabolism. Specifically, GLU and TG exhibited negative correlations with most key BAs, except for α-MCA. Conversly, BHBA and NEFA showed positive correlations, except for UDCA and α-MCA. Regarding inflammation parameters, only CP present negative correlations with three key BAs such as UDCA significantly, while HPT tended to correlate with UDCA and HDCA. Total PBAs and CDCA tended to correlate with OSI (p = 0.09; p = 0.06). SOD is significantly positively correlated with TCA (p = 0.04), and shows a positive correlation trend with total PBAs (p = 0.05) and GCA (p = 0.05). GSH-Px shows a significant positive correlation trend with total SBAs (p = 0.07), DCA (p = 0.09). MDA exhibited positive correlations with most key BAs, except for UDCA, TCA, HDCA and α-MCA.

### Dynamics of fecal microbiome and associations with bile acids during transition period

**Figure 4:**
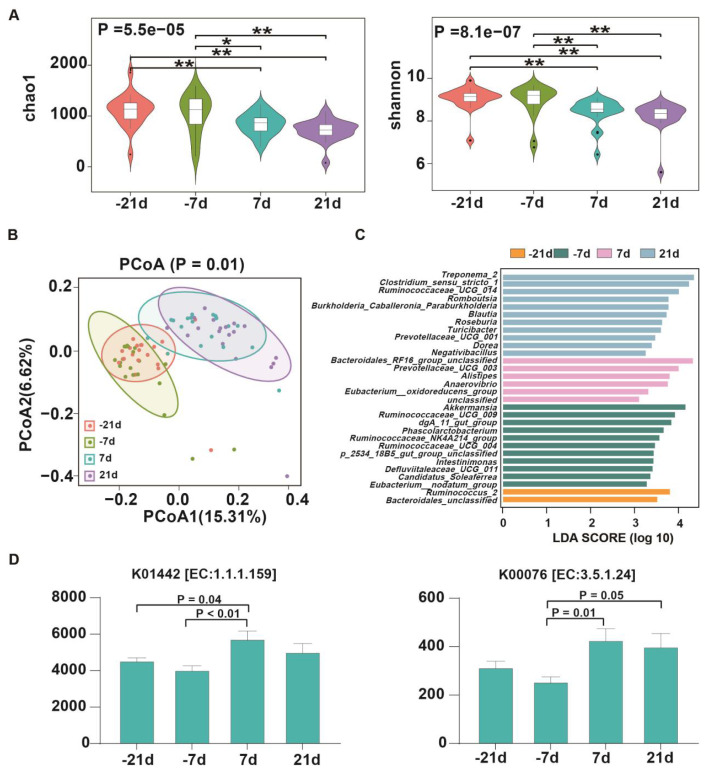
The dynamic changes of the fecal microbiota of 20 cows at d −21, −7, 7, and 21 relative to calving and its roles in bile acid changes. (A) The alpha diversity (Chao1 and Shannon) indices of fecal microbiota at four time points. (B) Comparison of beta diversity index (Bray-Curtis) of 20 dairy cows’ fecal microbiota at d −21, −7, +7, +21. (C) The significant biomarker genera at d −21, −7, 7, and 21 respectively which analyzed by linear discriminant analysis effect size. The p<0.05 and LDA>3 were presented. (D) Based on PICRUSt2 functional prediction. Gene copy numbers of 7α-hydroxysteroid dehydrogenase (7α-HSDH) and bile acid hydrolase (BSH) at d −21, −7, 7, and 21. * p<0.05, ** p<0.01. PCoA, principal coordinates analysis; KO1442, choloylglycine hydrolase; K00076, 7-alpha-hydroxysteroid dehydrogenase; LDA, linear discriminant analysis.

A total of 5,500,172 raw reads were obtained from the sequencing data of 79 fecal samples collected from 20 dairy cows at four peripartum time points (−21 d, −7 d, and +21 d: N = 20; +7d : N = 19). Following quality control, 22,749 ASVs were assigned from these reads. The results revealed that Chao1 and Shannon indices at −21 d and −7 d were significantly higher than at +7 d and +21 d (p<0.001), while −21 d did not differ significantly from −7 d (Figure 4A). Moreover, microbial β-diversity among the four time points exhibited significant differences (Figure 4B, p = 0.001).

**Figure 5:**
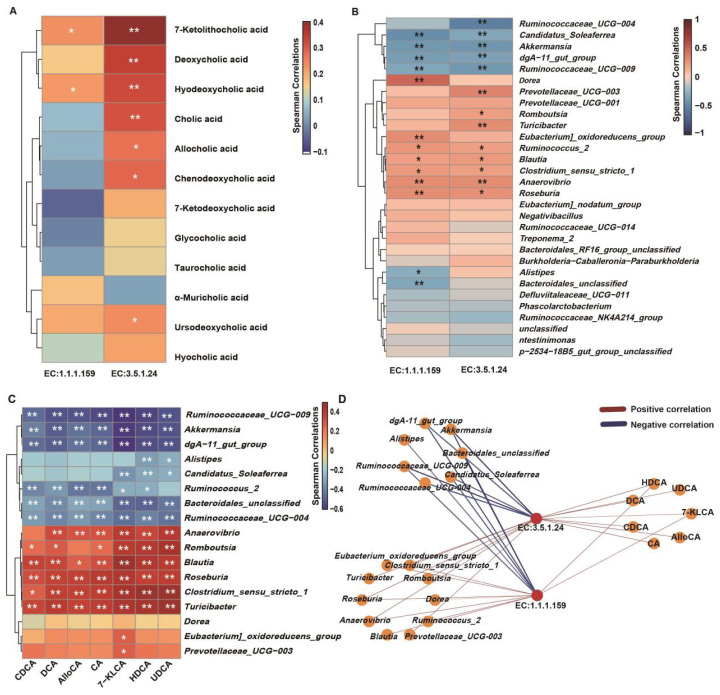
Spearman correlations among the 7α-hydroxysteroid dehydrogenase (7α-HSDH), bile acid hydrolase (BSH), bile acids that with VIP>1 and the biomarker genus at d −21, −7, +7, +21. (A) The correlations between 7α-HSDH, BSH and key bile acids. (B) The correlations between 7α-HSDH, BSH and key genus. (C) The correlations between bile acids that showed significant correlations in (A) and genus that that showed significant correlations in (B). (D) The integrated spearman correlations between bile acids and key eneyzms and significant different genus. * p<0.05, ** p<0.01. CDCA, chenodeoxycholic acid; DCA, deoxycholic acid; AlloCA, allocholic acid; CA, cholic acid; 7-KLCA, 7-ketolithocholic acid; HDCA, hyodeoxycholic acid; UDCA, ursodeoxycholic acid; VIP, variable importance projection.

In addition, a total of 30 significantly different genera (p<0.01, linear discriminant analysis [LDA]>3) were identified throughout the transition period. Among these genera, *Bacteroidales_unclassified* and *Ruminococcus_2* were regarded as biomarkers at −21 d; eleven genera, such as *Ruminococcaceae_NK4A214_group* and *dgA_11_gut_group*, were significantly higher at −7 d. At +7 d and +21 d, six and eleven biomarkers at the genus level were identified, respectively, with *Eubacterium_oxidoreducens_group*, *Treponema_2*, *Clostridium_sensu_stricto_1*, and *Ruminococcaceae_UCG_014* showing higher relative abundances (Figure 4C). Functional prediction using PICRUSt2 revealed significant differences (p<0.05) in functions related to SBA biosynthesis between prepartum and postpartum (Figure 4D). Specifically, K01442 (choloylglycine hydrolase, EC:3.5.1.24) was significantly higher at +7 d compared to −21 d (p = 0.04) and −7 d (p<0.01), while K00076 (7-alpha-hydroxysteroid dehydrogenase [7α-HSDH], EC:1.1.1.159) showed significantly higher levels at +7 d (p = 0.01) and +21 d (p = 0.05) compared to −7 d. The correlations between microbial functions and key BAs are shown in Figure 5A. 7-KLCA and HDCA, exhibited positive correlations with EC:1.1.1.159 and EC:3.5.1.24 significantly (p< 0.05). DCA, CA, AlloCA, CDCA, and UDCA, exhibited positive correlations with EC:3.5.1.24 significantly (p<0.05). A total of 17 biomarker genera, including 7 and 10 genera such as *Anaerovibrio* and *Ruminococcaceae_UCG_009* identified in Figure 4C, showed significant negative and positive correlations with EC:1.1.1.159 and EC:3.5.1.24, respectively (Figure 5B), and these genera also correlated with the seven BAs except for *Dorea* (Figure 5C). Consequently, a total of 17 important genera, 2 BA biosynthetic enzyme, and 7 key BAs were identified as playing crucial roles in BA metabolism during the transition period in dairy cows (Figure 5D).

## DISCUSSION

The postpartum period in dairy cattle is characterized by significant metabolic changes, including alterations in endocrine hormones, glucose, and lipid metabolism. Among these, the pronounced lipid metabolism, resulting from energy imbalance, serves as the primary contributor to various metabolic disorders during this phase. BA play a crucial role in lipid digestion, absorption, and metabolism [7]. However, there is a paucity of research on dynamic changes in BA levels in transition dairy cows. This study aims to explore the dynamic changes in peripheral plasma BA levels in dairy cows during the transition period and analyze their associations with basic physiological metabolism. Additionally, the role of gut microbiota in this process was investigated. These findings provide fundamental insights into BA metabolism in transitional dairy cows and contribute to the development of strategies for healthy feeding practices in dairy cows.

The peripheral concentrations of PBAs and SBAs are all significantly elevated at postpartum, with an increase exceeding nearly twofold. This result could be considered a metabolic adaptation of dairy cows to cope with various physiological changes in transition period. The outcome is comprehensible easily, the first explanation is that the postpartum diets for dairy cows typically contain higher fat and energy content. Additionally, the NEB of postpartum cows is more serious and the body fat is mobilized to meet the energy requirements of milking [29]. Consequently, the lipid metabolism is heightened in postpartum dairy cows. Consistent with this, the concentrations of NEFA and BHBA in this study showed a positive correlation with BA concentration, while glucose and TG were correlated negatively, indicating that body fat mobilization and lipid metabolism caused by NEB during perinatal period would promote the production of BA. Our previous study found that the BA concentration was significantly higher in the postpartum cows that with higher plasma NEFA concentration compared to thatcows with lower NEFA [22]. Currently, Dicks et al [30] reported the higher BAs content in postpartum cows and showed strong correlations with body scores and lipid mobilization. These results corroborate our experimental findings and support our hypothesis that postpartum cows require more BAs to adapt to the intense lipid metabolism.

The homeostasis of peripheral BAs depends on the regulations of liver and gut microbiome [6]. Previous research have shown a significant increase in the “Bile secretion” pathway in postpartum cows compared to the prepartum cows through liver the transcriptome analysis, although they did not focus on this topic [31,32]. Although this study lacked liver tissue data, the significant correlation between liver function biomarkers, especially AST, and total BAs and key BAs partially explains this phenomenon.

The gastrointestinal microbiome play important roles in the growth and development of calves [33] as well as the shape of traits in dairy cows [34]. Here, we investigated the response of the intestinal microbiome to the increased demand for BAs in transitional dairy cows. Consistent with the increase in BA concentration postpartum, the relative abundance of certain bacteria genus enriched with BA metabolism genes was significantly increased, for instance, the genus *Alistipes*. Lin et al [35] found the bile salt hydrolase (BSH)-carrying metagenome-assembled genomes predominately assigned to genus *Alistipes* by using the genome-centric approach in dairy cows. Zhuge et al [36] reported the positive correlation between BAs and *Alistipes*. In addition, the relative abundance of *Ruminococcaceae_UCG-014* and *Negativibacillus* were also higher in postpartum, and they belong to the BSH and 7α-HSDH-active family *Ruminococcaceae*, indicating they may have the ability to convert some PBAs into SBAs [37,38]. Consistent with the changes of bacterial genus, the abundance of two microbial enzymes, BSH and 7α-HSDH which involved in SBA biosynthesis were identified significantly increased in the postpartum dairy cows based on PICRUSt2 functional prediction. Taken together, these results suggest that both the liver and gut microbiota of dairy cows undergo functional changes to adapt to the increased demand for BAs during the transition period.

Inflammation and oxidative stress are major challenges suffered by perinatal dairy cows [39]. The UDCA, an SBA, is widely used in the treatment of cholestatic liver diseases [40], and has been shown to alleviate intestinal inflammation in neonatal dairy calves [41], early-weaned lambs [42], and piglets [20]. Additionally, certain BAs, such as TCA, have been reported to alleviate oxidative stress [43]. Consistent with these findings, our study observed a negative correlation between BA concentrations and inflammatory markers, as well as oxidative stress indicators. Our previous study has been revealed the links between BAs and immune-suppression of monocyte in transitional dairy cows [22]. Theses results suggest that the increased BAs may have benefits in alleviating inflammation and oxidative stress and improving the health of perinatal dairy cows. Future research should focus on systematically investigating the impact of BAs on the health of dairy cows, which could yield novel nutritional regulatory strategies for optimal management of dairy cows in transition period.

## CONCLUSION

In conclusion, this study unveiled the dynamic changes in peripheral BAs in dairy cows during the transition period. Our findings indicate a significant increase in BA concentrations postpartum, which positively correlate with indicators of energy metabolism such as NEFA and BHBA and oxidative stress markers, while negatively correlating with inflammation. Key BAs, including UDCA, were identified as significant contributors to these dynamics. We further explored the adaptation of the intestinal microbiome to BAs, revealing significant increases in the relative abundance of BSH and 7A- HSDH in postpartum stage. Importantly, we identified key genera, such as *Alistipes* and members of the *Ruminococcaceae* family, as pivotal players in this process. These findings underscore a heightened demand for BAs to accommodate robust lipid metabolism after calving in dairy cows, suggesting potential regulatory effects of BAs on dairy cow health. Future studies should systematically investigate the effects and regulatory mechanisms of supplementing key BAs or bile salt additives on the performance and health of transitional dairy cows. Such endeavors hold promise for offering novel perspectives and strategies to promote the healthy feeding of perinatal dairy cows.

## SUPPLEMENTARY MATERIAL

**Supplement 1**. Ingredients of the basal diet (%, DM basis)

**Supplement 2**. Dynamic profile and concentrations of the plasma bile acids in dairy cows

**Supplement 3**. Dynamic proportions of the plasma bile acids in dairy cows
