# Supplementary material for: Longitudinal dynamics of plasma bile acids and their associations with physiological parameters and fecal microbiome during the transition period in dairy cows
Source: Anim Biosci. 2025 Feb 27;38(6):1194–205. doi: 10.5713/ab.24.0628 (PMC12061570; doi:10.5713/ab.24.0628)
Supplement: Supplementary file 1 [file ab-24-0628-Supplementary-1.pdf]

**Supplement 1. Ingredients of the basal diet (%, DM basis)**

| Item | Dry period | Lactation period |
|---|---|---|
| **Ingredients** | | |
| Alfalfa | | 14.1 |
| Corn silage | 28 | 19.8 |
| Corn grain, ground | 15.4 | 15.2 |
| Soybean meal | 10.3 | 17.7 |
| Steam-flaked corn | | 12.7 |
| Sugar beet pulp | | 6.97 |
| Dicalcium phosphate | 0.12 | 0.51 |
| Sodium bicarbonate | 0.5 | 1.01 |
| Limestone, ground | | 0.76 |
| Fatty acid calcium | | 1.5 |
| Mycotoxin binder | | 0.1 |
| Wheat bran | 6.85 | |
| Rice straw | 17.9 | |
| Beer grains | | 1.43 |
| Oat hay | 19.9 | 7.05 |
| Yeast | | 0.1 |
| Salt | 0.17 | 0.51 |
| Premix[1] (dry period) | 0.86 | |
| Premix[2] (lactation period) | | 0.56 |
