# Supplementary material for: Longitudinal dynamics of plasma bile acids and their associations with physiological parameters and fecal microbiome during the transition period in dairy cows
Source: Anim Biosci. 2025 Feb 27;38(6):1194–205. doi: 10.5713/ab.24.0628 (PMC12061570; doi:10.5713/ab.24.0628)
Supplement: Supplementary file 2 [file ab-24-0628-Supplementary-2.pdf]

Supplement 2. Dynamic profile and concentrations of the plasma bile acids in dairy

	-21d	-7d	7d	21d	SEM	P-value
**Primary BA (ng/ml)**						
TCA	19292	38173	90961	50446	10535	< 0.01
CA	20941	18120	48188	49459	4410.61	< 0.01
GCA	13926	17640	34686	38063	4141.47	< 0.01
GCDCA	6227	7303	12144	13505	1496.89	< 0.01
CDCA	1672	2030	5252	6609	769.28	< 0.01
TCDCA	1263	1541	2467	1349	333.53	0.07
α-TMCA/β -TMCA	17.3	22.8	28.4	14.7	3.2461	0.02
β-MCA	10	9.8	10.8	11	0.5542	0.27
α-MCA	1.58	0.93	1.36	2.4	0.3802	0.05
Totally	81528	93330	224958	193345	19825	< 0.01
**Secondary BA (ng/ml)**						
MCA/HCA	10908	7811	20187	25096	2324.63	< 0.01
GDCA	5862	6875	10601	12877	1302	< 0.01
TDCA	4139	4924	10284	6288	11502	< 0.01
DCA	1975	1665	6882	5683	899	< 0.01
7-KDCA	902	920	6412	4652	1008	< 0.01
GLCA	969	934	904	1214	102	0.07
TLCA	1022	764	1090	782	163	0.3
AlloCA	611	544	1657	1494	175.01	< 0.01
TUDCA	748	653	690	646	66.6539	0.53
THDCA	350	345	356	339	35.5	0.97
HDCA	104	94.2	224	354	35.7	< 0.01
isoLCA	106	98.8	150	169	11.9	< 0.01
7-KLCA	72.9	63	183	167	22.1	< 0.01
LCA	86.8	79	126	146	11	< 0.01
UDCA	62.5	35.1	98.5	203.2	20.1	< 0.01
ApoCA	68.6	69.8	86.7	94.1	6.6407	0.02
3-DHCA	31.7	31.2	48.6	50.2	2.8098	< 0.01
12-KLCA	57.8	55.8	85.3	88.5	6.49	< 0.01
GHDCA	21.7	22.6	23.4	21.9	0.95	0.5
THCA	18.1	21.3	19.9	17.7	1.2335	0.05
MoCA	15.7	15.3	23.8	21.5	2.58	0.07
GUDCA	12.6	21.9	22.6	14.8	4.15	0.12
ω-MCA	11.7	11.6	11.9	11.7	0.44	0.46
Totally	16723	18833	40379	38086	3426	< 0.01
