# Supplementary material for: Longitudinal dynamics of plasma bile acids and their associations with physiological parameters and fecal microbiome during the transition period in dairy cows
Source: Anim Biosci. 2025 Feb 27;38(6):1194–205. doi: 10.5713/ab.24.0628 (PMC12061570; doi:10.5713/ab.24.0628)
Supplement: Supplementary file 3 [file ab-24-0628-Supplementary-3.pdf]

Supplement 3. Dynamic proportions of the plasma bile acids in dairy cows

	-21d	-7d	7d	21d	SEM	P-value
**Primary BA (%)**						
TCA	21.1	34.4	35.8	22.9	3.02	0.02
CA	22.9	16.3	19	22.5	1.93	0.15
GCA	15.2	15.9	13.7	17.3	1.1	0.3
GCDCA	6.81	6.59	4.78	6.14	0.46	< 0.01
CDCA	1.83	1.83	2.07	3.01	0.33	0.22
TCDCA	1.38	1.39	0.97	0.61	0.17	0.03
α-TMCA/β -TMCA	0.02	0.02	0.01	0.01	0.05	0.29
β-MCA	0.01	0.01	0	0	0.07	0.41
α-MCA	0	0	0	0	0.08	0.48
Totally	81.9	82.2	84.9	84.7	3.7	0.86
**Secondary BA (%)**						
MCA/HCA	11.9	7.04	7.95	11.4	1.23	0.02
GDCA	6.41	6.2	4.18	5.86	0.41	< 0.01
TDCA	4.52	4.44	4.05	2.86	0.37	0.02
DCA	2.16	1.5	2.71	2.58	0.32	0.03
7-KDCA	0.99	0.83	2.53	2.12	0.35	< 0.01
GLCA	1.06	0.84	0.36	0.55	0.07	< 0.01
TLCA	1.12	0.69	0.43	0.36	0.15	0.06
AlloCA	0.67	0.49	0.65	0.68	0.08	0.73
TUDCA	0.82	0.59	0.27	0.29	0.08	< 0.01
THDCA	0.38	0.31	0.14	0.15	0.05	< 0.01
HDCA	0.11	0.08	0.09	0.16	0.03	0.02
isoLCA	0.12	0.09	0.06	0.08	0.01	< 0.01
7-KLCA	0.08	0.06	0.07	0.08	0.13	0.58
LCA	0.09	0.07	0.05	0.07	0.01	< 0.01
UDCA	0.07	0.03	0.04	0.09	0.06	0.41
ApoCA	0.07	0.06	0.03	0.04	0.08	0.57
3-DHCA	0.03	0.03	0.02	0.02	0.003	0.21
12-KLCA	0.06	0.05	0.03	0.04	0.08	0.53
GHDCA	0.02	0.02	0.01	0.01	0.08	0.44
THCA	0.02	0.02	0.01	0.01	0.08	0.43
MoCA	0.02	0.01	0.01	0.01	0.1	0.4
GUDCA	0.01	0.02	0.01	0.01	0.08	0.5
ω-MCA	0.01	0.01	0	0.01	0.08	0.41
Totally	18.1	17.8	15.1	15.3	0.9	0.02
